# Integrating Study on Qualitative and Quantitative Characterization of the Major Constituents in Shuanghuanglian Injection with UHPLC/Q-Orbitrap-MS and UPLC-PDA

**DOI:** 10.1155/2021/9991363

**Published:** 2021-05-21

**Authors:** Gen Xue, Meijuan Zhu, Ning Meng, Jianli Guan, Jide Zhang, Jing Yang, Yuefei Wang, Ying Cui, Xin Chai

**Affiliations:** ^1^State Key Laboratory of Component-Based Chinese Medicine, Tianjin University of Traditional Chinese Medicine, Tianjin 301617, China; ^2^Tianjin Key Laboratory of TCM Chemistry and Analysis, Tianjin University of Traditional Chinese Medicine, Tianjin 301617, China; ^3^Henan Fusen Pharmaceutical Co. Ltd., Nanyang 474450, China

## Abstract

As a clinically effective traditional Chinese medicine injection (TCMI), Shuanghuanglian injection (SHLI) is widely used in the treatment of upper respiratory tract infection and pneumonia. However, the shortage of quality analysis is a limitation that remains to be improved in the clinical application of SHLI. In this study, taking advantage of ultra-high-performance liquid chromatography tandem Q-Exactive Orbitrap high-resolution mass spectrometry (UHPLC/Q-Orbitrap-MS), 31 chemical components (eight organic acids, eleven flavonoids, five iridoid glycosides, four phenylethanoid glycosides, and three lignans) in SHLI were characterized, among which 22 components were unambiguously identified by reference compounds. The brief prediction results of network pharmacology indicated that the 22 targeted components may have anti-inflammatory, antibacterial, antiviral, and immunomodulatory activities. Using multiwavelength switching method, the 22 targeted components were quantified by ultra-performance liquid chromatography with photodiode array detector (UPLC-PDA) after the methodological validation. Based on the successfully established method, the total content of 22 components in 20 batches of SHLIs was efficiently determined with a slight variation between 10.25 and 11.28 mg/mL, which accounted for 38.7% in total solid of SHLI. This study performed a reliable chemical identification and provided a rapid and effective method for quality analysis, which contributed to the in-depth investigation and application of SHLI.

## 1. Introduction

Traditional Chinese medicine (TCM) has been widely utilized in clinical practice across Asia and gradually attracts more attention in Western countries for its reliable efficacy [1]. As an important innovation of the modernization of TCM, traditional Chinese medicine injections (TCMIs) have the advantages of rapid onset, targeted drug delivery, high bioavailability, and so on [2]. Based on the above perceptions, TCMIs play a pivotal role in the treatment of cardiovascular and cerebrovascular diseases, respiratory diseases, and tumors [3–6]. As the complex chemical components and the feature of direct intravenous injection restrict the clinical application of TCMIs, the research on their quality and chemical composition has attracted the attention of numerous researchers and regulators.

As an important TCMI prepared by *Flos Lonicerae japonicae,Fructus Forsythiae*, and *Radix Scutellariae* through modern technology, SHLI is commonly used to treat upper respiratory tract infection, pneumonia, and fever [7–9]. SHL preparations involve flavonoids, phenolic acids, phenolic glycosides, and other ingredients, which exhibit obvious antibacterial, antiviral, and immune-enhancement activities proved by modern pharmacological studies [10–14]. Qualitative and quantitative analyses of SHL preparations have been reported previously. Based on UPLC/Qtof MS^E^ with UNIFI informatics platform, 170 components were characterized in SHL oral liquid, 44 of which were further determined by reference compounds [10]. Wang et al. simultaneously quantified 22 active compounds in SHL preparations (including capsule, granule, and oral liquid) by HPLC-DAD [15]. Up to now, the researches of SHL for injection mainly focused on SHL powder injection (SHLPI). Sun et al. unambiguously or tentatively identified 125 compounds of SHLPI by HPLC-ESI-IT-TOF-MS [16]. Li et al. developed an HPLC method for the simultaneous analysis of baicalin and other 14 compounds to evaluate the quality consistency of SHLPI [17]. By comparison, the relevant reports of SHLI were relatively limited. Only the content of chlorogenic acid, baicalin, and pillyrin is prescribed in the current ministry standard; hence, the comprehensive research on quality control of SHLI is more urgent.

To ensure medication safety for the public, the National Medical Products Administration launched the safety re-evaluation of TCMIs in 2009. Thereinto, clarification of chemical compositions and improvement of the quality evaluation method are the critical problem. The selection of index components is the prerequisite issue. Network pharmacology is based on high-throughput omics data analysis and network database retrieval, which has become a popular tool in the field of TCM research. As a compatible method to predict the complex and holistic mechanism of TCM, network pharmacology has unique advantages in analysing the active ingredients and illustrating the molecular mechanism [18]. The network pharmacology could build a bridge to study the relationship between qualitative and quantitative analysis, which conduces to identifying the index compounds. With this strategy, now we mainly focus on the secondary metabolites in SHLI to carry out qualitative and quantitative characterization with the aid of network pharmacology.

In this study, 31 chemical components (eight organic acids, eleven flavonoids, five iridoid glycosides, four phenylethanoid glycosides, and three lignans) in SHLI were characterized by UHPLC/Q-Orbitrap-MS. The 22 compounds identified by reference compounds were found to have anti-inflammatory, antibacterial, antiviral, and immune-enhancing activities by the strategy of network pharmacology, which was associated with SHLI function. By taking the great disparity of chemical constituents' contents into account, the proportional dilution method was employed to quantify the 22 targeted components in 20 batches of SHLIs through UPLC-PDA multiwavelength switching method. Also, the lot-to-lot consistency was assessed among different batches. In short, the established method improved the quality control for SHLI and provided scientific evidence for the clinical application of SHLI.

## 2. Materials and Methods

### 2.1. Reagents and Materials

LC-grade methanol and acetonitrile were purchased from Fisher Scientific (Fair Lawn, NJ, USA). Formic acid and dimethyl-sulfoxide (DMSO) were acquired from Shanghai Aladdin Biochemical Technology Co., Ltd. (Shanghai, China) and Damao Chemical Reagent Factory (Tianjin, China), respectively. Water used in the experiment was purified by Milli-Q water purification system (Millipore, Billerica, MA, USA). Twenty batches of SHLIs (20 mL/per ampoule) produced in 2018 and 2019 were provided by Henan Fusen Pharmaceutical Co., Ltd. (Henan, China) and numbered as S1–S20.

Chlorogenic acid (Cha, >96.80%), caffeic acid (Caa, >99.70%), scutellarin (Scu, >91.70%), forsythoside A (FoA, >97.20%), pillyrin (Pil, >95.10%), baicalein (Bai, >97.90%), oroxindin (Oro, >98.00%), and wogonin (Won, >97.00%) were obtained from National Institutes for Food and Drug Control (Beijing, China). Secoxyloganin (Seg, >98.00%) was purchased from Chengdu Dexter Biotechnology Co., Ltd. (Sichuan, China). Neochlorogenic acid (Nea), forsythoside E (FoE), cryptochlorogenic acid (Cra), secologanic acid (Sea), isoforsythiaside (Iso), hyperoside (Hyp), luteolin-7-*O*-*β*-D-glucoside (Lug), isochlorogenic acid B (IaB), isochlorogenic acid A (IaA), isochlorogenic acid C (IaC), chrysin-7-*O*-*β*-D-glucuronide (Chg), oroxylin A-7-*O*-*β*-D-glucuronide (Org), baicalin (Bac), and oroxylin A (OrA) with purity above 98% were acquired from Shanghai Yuanye Bio-Technology Co., Ltd. (Shanghai, China).

### 2.2. Preparation of Reference and Sample Solutions

Twenty-three reference compounds were accurately weighed and respectively dissolved in methanol aqueous solution as stock solutions except for Bac dissolved by DMSO. Except Bac, each stock solution was accurately measured and then placed in a 25 mL volumetric flask and diluted with 10% methanol aqueous solution (*v*/*v*), which was employed to prepare a mixed stock solution at a final concentration of 32.77 *μ*g/mL Nea, 100.0 *μ*g/mL FoE, 38.96 *μ*g/mL Cha, 37.73 *μ*g/mL Cra, 6.048 *μ*g/mL Caa, 32.00 *μ*g/mL Sea, 18.00 *μ*g/mL Seg, 74.66 *μ*g/mL Iso, 6.636 *μ*g/mL Hyp, 11.60 *μ*g/mL Scu, 77.40 *μ*g/mL FoA, 16.72 *μ*g/mL IaB, 6.000 *μ*g/mL IaA, 16.83 *μ*g/mL IaC, 27.81 *μ*g/mL Pil, 22.04 *μ*g/mL Chg, 56.00 *μ*g/mL Org, 4.624 *μ*g/mL Oro, 10.12 *μ*g/mL Bai, 0.9744 *μ*g/mL Won, and 0.8260 *μ*g/mL OrA, respectively. In order to plot the calibration curves, six working standard solutions at the different concentrations were achieved by serially diluting the mixed stock solution with 10% methanol aqueous solution. Similarly, the individual stock solution of Bac at a concentration of 0.12 mg/mL and a series of working solutions were obtained.

SHLI was diluted 25 times with 10% methanol aqueous solution for UHPLC-MS/MS analysis and quantitative analysis of the compounds except Bac. For Bac quantification, SHLI was diluted 250 times with 10% methanol aqueous solution. All solutions were stored at 4°C until analysis.

### 2.3. UHPLC-MS/MS Conditions for Qualitative Analysis

The qualitative analysis was carried out on a Thermo Scientific UltiMate 3000 instrument (Thermo Fisher Scientific, San José, CA, USA) coupled with a *Q* Exactive Orbitrap MS equipped with heated electrospray ionization (HESI) source. Using an ACQUITY UPLC^®^ HSS T3 column (2.1 × 100 mm, 1.8 *μ*m, Waters, Milford, MA, USA), the separation was performed with mobile phase composed of 0.1% formic acid aqueous solution (*v*/*v*) (A) and acetonitrile (B) in a gradient elution at 50°C. The optimized gradient elution was performed as follows: 0−7 min, 3−13% B; 7−18 min, 13−27% B; 18−20 min, 27%–48% B; 20−21 min, 48−90% B; and 21−24 min, 90% B. The flow rate and injection volume were 0.3 mL/min and 2 *μ*L, respectively. Applying the Full MS and dd-MS^2^ (TopN) modes in both positive and negative ion modes, the sample was analysed in the range of *m*/*z* 150−2000 Da at the resolution of 70000 FWHM in full-scan MS and 17500 FWHM in MS^2^ mode. The optimal MS conditions were as follows: sheath gas flow rate (N_2_), 35 Arb; aux gas flow rate (N_2_), 10 Arb; spray voltage, 3.5 kV; aux gas heater temperature, 350°C; and capillary temperature, 350°C. All the data were acquired and processed by the Xcalibur™ 4.1 software (Thermo Fisher Scientific).

### 2.4. UPLC-PDA Conditions for Quantitative Analysis

An ACQUITY UPLC I-class system (Waters), equipped with a binary solvent manager, an autosampler, and a PDA detector, was used to perform the quantitative analysis. Multiwavelength switching method was adopted on the PDA detector, and timing wavelength switching was employed as follows: 0.00–5.00 min, 327 nm; 5.00–5.60 min, 280 nm; 5.60–6.12 min, 325 nm; 6.12–8.50 min, 240 nm; 8.50–9.50 min, 235 nm; 9.50–9.80 min, 240 nm; 9.80–10.00 min, 250 nm; 10.00–14.00 min, 327 nm; 14.00–14.50 min, 254 nm; 14.50–17.65 min, 276 nm; and 17.65–25.00, 270 nm. Other liquid chromatography parameters were the same as in the qualitative analysis described above.

### 2.5. Network Pharmacology Analysis

The 2D chemical structures of the targeted compounds were achieved from the PubChem database (https://pubchem.ncbi.nlm.nih.gov/) [19]. Based on the reverse pharmacophore matching strategy, the SwissTargetPrediction database (http://www.swisstargetprediction.ch/) was used for predicting the potential molecular targets [20]. The related pathways of key targets were obtained by the Kyoto Encyclopedia of Genes and Genomes (KEGG) pathway enrichment analysis through the DAVID 6.8 database (https://david.ncifcrf.gov/home.jsp) [21]. The KEGG database (https://www.kegg.jp/) could identify the potential activities related with candidate pathways. An interactional and visualized network of “decoction pieces−TCMI−compounds−targets−signaling−pathways−pharmacological activity” was constructed by Origin 9.6 software.

### 2.6. Methodological Validation of the Quantitative Analysis

The quantitative analysis established in this study was validated for linearity, limit of detection (LOD), limit of quantitation (LOQ), precision (intra- and interday), stability, repeatability, and recovery test. The calibration curves were obtained by fitting the peak area (*y*) of the analytes and the corresponding concentrations (*x*) in duplicate. Based on the reference solutions, the LOD and LOQ were determined at the signal-to-noise ratio of 3 and 10, respectively. The intra- and interday precisions were calculated by analysing six repetitive injections in the single day and in three consecutive days, respectively. The sample solution, stashed in the autosampler at 10°C, was analysed by replicate injection at 0, 2, 4, 6, 8, 10, and 12 h, respectively. Verification of repeatability was constructed on six different samples in duplicate. The recovery test was estimated by analysing six sample solutions that were prepared by adding the corresponding amount of standard solutions into precisely measured 0.5 mL injections.

### 2.7. Data Analysis

The heat map, box-plot, parallel coordinate plot, and bar graph were performed using Origin 2019 software (OriginLab Ltd., Northampton, MA, USA).

## 3. Results and Discussion

### 3.1. Characterization of Chemical Constituents in SHLI by UHPLC/Q-Orbitrap-MS

**Figure 1:**
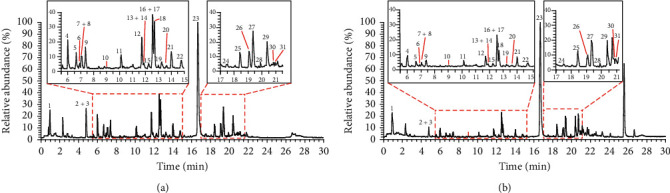
Total ion chromatograms of SHLI in positive ion mode (a) and negative ion mode (b).

To characterize the chemical constituents in SHLI, a UHPLC/Q-Orbitrap-MS method was established. Total ion chromatograms of SHLI in positive and negative ion modes were shown in Figures 1(a) and 1(b), respectively. By comparing with the reference compounds and characteristic MS fragmentation pattern, a total of 31 components were identified or tentatively characterized, including eight organic acids, eleven flavonoids, five iridoid glycosides, four phenylethanoid glycosides, and three lignans. Among them, 22 components were unquestionably validated with the reference compounds. The information of all the compounds was summarized in Table 1.

Seg (peak 11) and Bac (peak 23) were chosen as two examples to describe the fragment behaviours of these compounds. As the representative of the iridoid glycosides, Seg has shown a quasimolecular ion at *m/z* 403.12305 [M−H]^−^ in the negative ion mode. The characteristic fragment ions at *m/z* 223.05956 [M−H−Glc]^−^, *m/z* 165.05382 [M−H−Glc−CH_2_COO]^−^, *m/z* 121.02790 [M−H−Glc−CH_2_COO–CO_2_]^−^, and *m/z* 95.04853 [M−H−Glc−CH_2_COO–CO_2_–C_2_H_2_]^−^ were attributed to the successive loss of glucose, CH_2_COO, CO_2_, and C_2_H_2_, which were basically consistent with the mass spectrometry fragmentation law of iridoid glycosides [16]. Bac was not only the flavonoid with the highest content in *Radix Scutellariae*, but also one of the main active ingredients in SHLI. In the negative ion spectrum of Bac, the dimer quasimolecular ion at *m/z* 891.16333 [2M−H]^−^ was observed. Meanwhile, due to high-energy collision, the quasimolecular ion at *m/z* 445.07593 [M−H]^−^ lost a glucuronic acid residue to generate the characteristic fragment ion at *m/z* 269.04449 [M−H−GlcAr]^−^ with the highest abundance [10]. As shown in Figures S1 and S2, the proposed fragmentation pathways of Seg and Bac in the negative mode were depicted, respectively.

**Figure 2:**
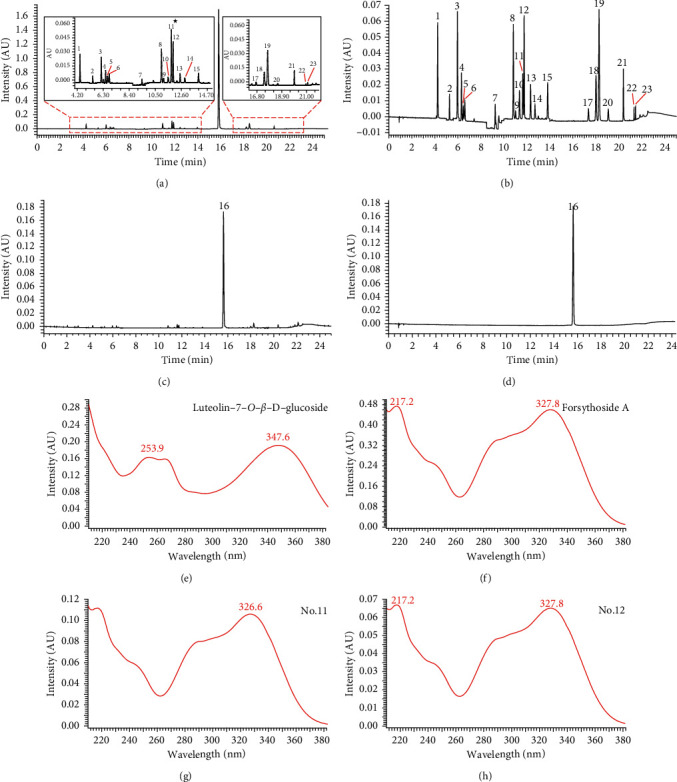
UPLC-PDA chromatograms of SHLI sample solution with low dilution ratio (a), the mixed standard solution (b), the SHLI sample solution with high dilution ratio (c) and the baicalin standard solution (d), the ultraviolet absorption curve of luteolin-7-*O*-*β*-D-glucoside (e), forsythoside A (f), no. 11 chromatographic peak (g), and no. 12 chromatographic peak (h).

In the UPLC-PDA chromatogram, we run into a noteworthy phenomenon that the retention time of no. 11 chromatographic peak was in accord with Lug, whereas its ultraviolet absorption curve was basically consistent with FoA (no. 12), as shown in Figure 2. Combined with the results of LC-MS analysis (peak 16, Lug, *t*_R_ = 12.50 min; peak 17, FoA isomer (FAi), *t*_R_ = 12.52 min), no. 11 peak displayed in Figure 2(a) was the overlap of FAi and Lug. Therefore, the mixture of FAi and Lug should not be considered as an index component for the quality control of SHLI in the follow-up studies.

### 3.2. Selection of Index Components with Network Pharmacology Strategy

It is difficult to evaluate the quality of TCM preparations using single-component evaluation model objectively and accurately with the development of TCM modernization. Based on network pharmacology, the study for quality control of TCM starts from the interaction between the chemical components and the disease, and then the “chemical composition-target-disease network” is constructed to obtain the core chemical components as the index compounds.

Aiming at the unequivocal 22 compounds identified by the reference compounds in the qualitative analysis with LC-MS, a network pharmacology research strategy was imitated to briefly predict the potential activities, which provided a basis for the selection of index components for the quality control of SHLI. The chemical structures of the 22 target compounds, achieved from the PubChem database, were uploaded to the SwissTargetPrediction to accurately predict bioactive molecular targets with *Homo* as a limited species. With the aid of DAVID and KEGG Pathway databases, the correlative pathways were enriched and analysed by selecting the condition “*Homo* species,” respectively.

**Figure 3:**
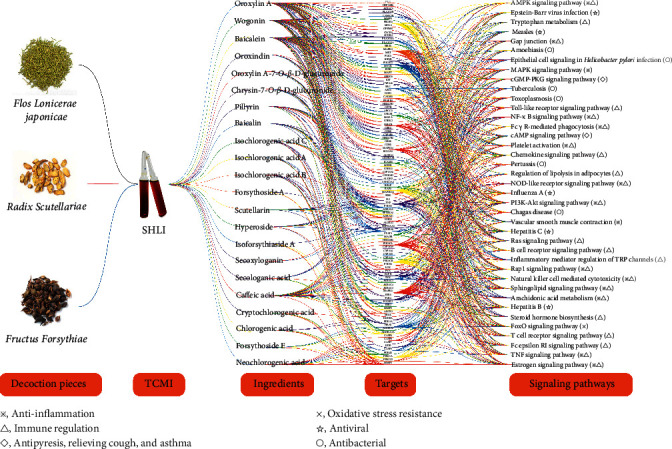
Parallel coordinate plot for the herbs-injection-compounds-targets-signaling pathways-pharmacological activity of SHLI.

Ultimately, 238 targets corresponding to the 22 active ingredients in SHLI were screened after eliminating duplicates. Through the KEGG Pathway enrichment analysis, a total of 93 pathways were acquired (*p* < 0.01), and after eliminating irrelevant pathways, only 39 correlative pathways were included in the analysis (the unabridged information was shown in Tables S1 and S2). Notably, the KEGG Pathway enrichment results revealed the close association with anti-inflammatory, antibacterial, antiviral, and immunomodulatory activities. For example, estrogen signaling pathway, TNF signaling pathway, T-cell receptor signaling pathway, and B cell receptor signaling pathway were closely related to anti-inflammatory and immunoreactive activities. Meanwhile, the antibacterial and antiviral activity could be explicitly unscrambled from influenza A and tuberculosis signaling pathways [24, 25]. All the predictions were highly consistent with the efficacy of SHLI in clearing heat and detoxifying, indicating the scientific nature and rationality of this study. The integrated network was illustrated in Figure 3.

### 3.3. Optimization of UPLC-PDA Chromatographic Conditions

The optimized chromatographic conditions could pave the way for methodological validation. Aiming at the better separation and sensitivity of the tested compounds, the chromatographic column, including ACQUITY HSS T3 column (2.1 × 100 mm, 1.8 *μ*m), BEH C18 column (2.1 × 50 mm, 1.7 *μ*m), and BEH C18 column (2.1 × 100 mm, 1.7 *μ*m), the mobile phase (acetonitrile − water containing formic acid and methanol − water containing formic acid), the program of gradient elution, the column temperature (30°C, 40°C, and 50°C), and detecting wavelength were systematically optimized. Eventually, the optimal separation was achieved on an HSS T3 column at 50°C with mobile phase composed of 0.1% formic acid aqueous solution − acetonitrile. Considering the difference in the maximum absorption wavelength of the different categories of compounds in SHLI, the multiwavelength switching method was adopted and described in the section of quantitative chromatographic condition.

In chromatogram, the response of Bac was so high that it masked the other components, which was not conducive to the accurate quantification of trace components. Another challenge we encountered was how to realize the simultaneous determination of components with the disparity in content in SHLI. The proportional dilution method was preferably employed to address the above issue. SHLI was diluted 25 times with 10% methanol aqueous solution for methodological validation and quantitative analysis of the 21 compounds, including Nea, FoE, Cha, Cra, Caa, Sea, Seg, Iso, Hyp, Scu, FoA, IaB, IaA, IaC, Pil, Chg, Org, Oro, Bai, Won, and OrA. Meanwhile, SHLI was diluted 250 times with 10% methanol aqueous solution for Bac.

### 3.4. Method Validation of UPLC-PDA Analysis for Quantitation of 22 Compounds in SHLI

**Figure 4:**
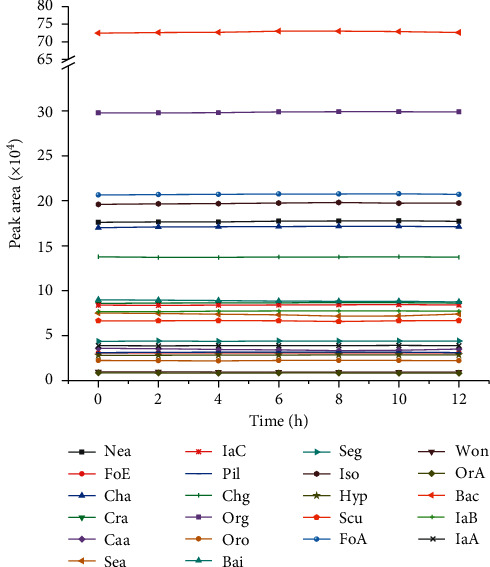
The stability study of tested compounds in sample solution during 12 h.

The favourable validation results of the simultaneous quantitative analysis of 22 compounds in SHLI were exhibited in Table 2. The linear relationship (*r* > 0.999) of the tested components was satisfactory within the corresponding concentration range. And the overall LOD and LOQ values for all analytes were less than 0.1736 and 0.5208 *µ*g/mL, respectively. In addition, the RSDs of the intra- and interday precisions, stability (Figure 4), and repeatability were proven below 3.5%. Finally, the range from 95.06% to 109.3% was obtained about the average recoveries, which indicated the method had well accuracy with RSD below 2.9%. All the above items demonstrated that the quantitative method could reliably pave the way for the simultaneous determination of the 22 targeted compounds in SHLI.

Due to the complex chemical composition of TCMIs, injections made from multicomponent should be quantitatively analysed as much as possible for the compounds with well-defined structures. Compared to the previously reported analytical methods (Table 3), the established method in our study exhibited advantages of shorter analytical time, higher resolution, and more tested compounds, which provided a better alternative for evaluating the quality of SHLI.

### 3.5. Visual Modes of the Quantitative Analysis Results of 22 Components in Different Batches of SHLIs

Plot-to-plot consistency evaluation is the foundational requirement for quality control to ensure the safe use of drugs. Through UPLC-PDA multiwavelength switching method coupled with proportional dilution method that verified by systematic methodology, the 22 targeted components were successfully quantified for 20 batches of SHLIs. As shown in Table S3, the total content of the tested components ranged from 10.25 to 11.28 mg/mL with RSD below 2.9%, which indicated no significant difference in the sum of targeted components from the different batches of SHLIs. However, the RSDs range of the different targeted components was 1.7–24.1%. Except for Bac with the high content, the RSDs for the rest of components were greater than 3.0%, manifesting that the content of each component in different batches varied to a certain extent.

**Figure 5:**
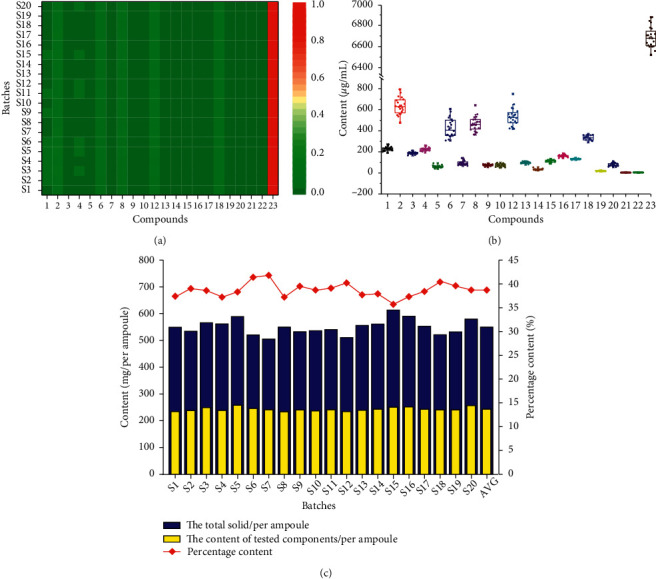
The heat map (a), the box-plot (b), and the bar graph (c) for the quantitative analysis of 22 targeted compounds in SHLIs of 20 batches.

In order to reveal the content variations of each targeted component in SHLI more intuitively, the normalized content of 22 compounds from 20 batches was used to plot the heat map, which mirrored the fluctuation of 22 compounds in different batches through the similarity of the same horizontal colour gradient, as shown in Figure 5(a). Among the 22 tested components, the colour depth of Nea, FoE, Cra, Sea, Seg, Iso, and FoA fluctuated greatly, indicating the content of these compounds varied highly among the different batches. Simultaneously, the box-plot was shown in Figure 5(b) and employed to reflect the dispersion of measured results for the focused secondary metabolites in 20 batches of SHLIs. Bac clearly exhibited a higher box compared with the others, indicating that Bac was present in higher content. And the above content results indicated that Bac accounts for 59.36–65.31% of all analytes in 20 batches.

The percentage of compounds with well-defined structures in the total solid should not be less than 60% for TCMIs made from multicomponent [31]. Thus, we wanted to calculate the total amount of 22 targeted compounds as a percentage of the total solid of SHLI. Different batches of SHLIs were poured into the evaporating dish and freeze-dried to determine the total solid. Calculation equation (1) was expressed as follows:

(1) $$\begin{array}{c} \text{PCT}=\frac{M_{t}}{M_{S}}\times 100\%. \end{array}$$

PCT is the mass percentage of the targeted compounds in total solid; *M*_*t*_ is the total content of the 22 targeted compounds per ampoule (20 mL); and *M*_*s*_ is the total solid of SHLI per ampoule (20 mL). As shown in Table S4 and Figure 5(c), the results manifested that the content of the 22 targeted components accounted for 38.7% of the total solid in the 20 batches of SHLIs produced in 2018 and 2019.

## 4. Conclusions

In this study, an accurate and reliable quality control method for SHLI was established. Thirty-one compounds in SHLI were unambiguously or tentatively identified by UHPLC/Q-Orbitrap-MS. Based on the qualitative analysis, a UPLC-PDA multiwavelength switching method coupled with proportional dilution method was established for quantitative analysis of 22 representative compounds in 20 batches SHLIs, which has been demonstrated to have anti-inflammatory, antibacterial, antiviral, and immunomodulatory activities. Results exhibited that it is reliable and applicable for quality control of SHLI and paved the way for the further application of SHLI clinically.

## Figures and Tables

**Table 1:** Characterization of chemical constituents of SHLI by UHPLC/Q-Orbitrap-MS (negative ion mode).

Serial no.	*t*_R_ (min)	Formula	Theoretical value (*m/z*)	Measured value (*m/z*)	Fragment ions (*m/z*) (%)	Error (ppm)	Identification [reference]
1	0.97	C_7_H_12_O_6_	191.05501	191.05478	191.05478 (100.00), 173.04419 (2.15), 127.03841 (4.55), 85.02785 (24.53)	−1.3	Quinic acid [16]
2	4.75	C_16_H_24_O_10_	375.12857	375.12862	213.07541 (75.49), 169.08653 (14.02), 151.07477 (29.82), 125.05906 (100.00)	0.2	Adoxosidic acid [22]
3^*∗*^	4.81	C_16_H_18_O_9_	353.08671	353.08652	191.05458 (100), 179.03343 (51.94), 173.04393 (4.23), 161.02283 (4.36), 135.04344 (64.31)	−0.5	Neochlorogenic acid
4^*∗*^	6.02	C_20_H_30_O_12_	461.16535	461.16486	205.07030 (17.21), 163.05919 (8.64), 135.04338 (80.33)	−1.1	Forsythoside E
5^*∗*^	6.55	C_16_H_18_O_9_	353.08671	353.08649	191.05450 (100.00), 179.03336 (1.07), 161.02281 (1.66), 135.04355 (1.19)	−0.6	Chlorogenic acid
6	6.90	C_16_H_22_O_11_	389.19784	389.10764	345.11707 (13.92), 209.04453 (8.56), 165.05368 (18.26)	−0.5	Secoxyloganic acid [16]
7^*∗*^	7.06	C_16_H_18_O_9_	353.08671	353.08659	173.04388 (100.00), 179.03334 (69.75), 135.04337 (79.89), 191.05444 (50.39), 161.02251 (4.37)	−0.5	Cryptochlorogenic acid
8^*∗*^	7.13	C_9_H_8_O_4_	179.03389	179.03349	135.04337 (100.00)	−2.2	Caffeic acid
9^*∗*^	7.29	C_16_H_22_O_10_	373.11292	373.11246	193.04903 (35.66), 179.05476 (4.45), 167.07019 (4.84), 149.05978 (25.30)	−1.2	Secologanic acid
10	9.06	C_18_H_28_O_12_	435.14970^#^	435.14932^#^	227.09137 (100.00)	−0.9	Loganin [16]
11^*∗*^	10.12	C_17_H_24_O_11_	403.1235	403.12305	223.05956 (14.49), 165.05382 (25.79), 121.02790 (100.00), 95.04853 (35.64)	−1.1	Secoxyloganin
12^*∗*^	11.58	C_29_H_36_O_15_	623.19705	623.19574	461.16406 (4.05), 179.03302 (10.81), 161.02254 (100.00), 135.04333 (19.30)	−2.1	Isoforsythiaside
13^*∗*^	11.74	C_21_H_20_O_12_	463.08710	463.08684	301.03384 (100.00)	−0.5	Hyperoside
14	11.89	C_27_H_30_O_16_	609.18140	609.17975	301.03247 (50.01), 300.02603 (100.00), 271.02350 (69.57), 255.02832 (33.46), 151.00186 (14.91)	−2.7	Rutin [23]
15^*∗*^	12.25	C_21_H_18_O_12_	461.07465	461.07092	285.03891 (6.58)	−1.2	Scutellarin
16^*∗*^	12.50	C_21_H_20_O_11_	447.09219	447.09143	285.03851 (100.00)	−0.7	Luteolin-7-*O*-*β*-*D*-glucoside
17	12.52	C_29_H_36_O_15_	623.19705	623.19537	461.16425 (1.43), 179.03326 (10.44), 161.02277 (100.00), 135.04337 (20.60), 133.02780 (31.88)	−2.6	Forsythoside A isomer
18^*∗*^	12.67	C_29_H_36_O_15_	623.19705	623.19556	461.16406 (4.05), 179.03302 (10.81), 161.02254 (100.00), 135.04333 (19.30), 133.02763 (23.50)	−2.5	Forsythoside A
19^*∗*^	13.22	C_25_H_24_O_12_	515.11840	515.11743	353.08606 (52.36), 191.05440 (48.98), 179.03325 (77.95), 173.04381 (100.00), 161.02251 (33.59), 135.04329 (87.51),	1.9	Isochlorogenic acid B
20^*∗*^	13.59	C_25_H_24_O_12_	515.11840	515.11780	353.08609 (52.38), 191.05447 (100.00), 179.03329 (52.09), 173.04396 (5.37), 161.02260 (5.92), 135.04335 (56.91),	−1.1	Isochlorogenic acid A
21	13.99	C_26_H_32_O_11_	519.18609	519.18536	357.13263 (62.44), 342.10983 (4.01), 136.014483 (68.79), 151.03835 (100.00)	−1.4	Pinoresinol-4-*O*-*β*-D-glucopyranoside [16]
22^*∗*^	14.75	C_25_H_24_O_12_	515.11840	515.11749	353.08636 (70.69), 191.05452 (39.63), 179.03331 (63.38), 173.04388 (100.00), 161.02257 (6.37), 135.04337 (60.85)	−1.3	Isochlorogenic acid C
23	16.44	C_21_H_18_O_11_	445.07654	445.07593	269.04449 (100.00)	−1.4	Baicalin [10]
24	17.52	C_27_H_34_O_11_	579.20722^#^	579.20630^#^	371.14874 (100.00), 356.12558 (68.45),	−1.6	Pillyrin isomer
25^*∗*^	18.43	C_27_H_34_O_11_	579.20722^#^	579.20605^#^	371.14896 (92.52), 356.12558 (100.00)	−2.0	Pillyrin
26^*∗*^	19.03	C_21_H_18_O_10_	429.08162	429.08075	253.04904 (100.00), 175.02341 (4.07)	−2.0	Chrysin-7-*O*-*β*-D-glucuronide
27^*∗*^	19.33	C_22_H_20_O_11_	459.09219	459.09134	283.05988 (79.01), 268.03647 (100.00), 175.02272 (5.69)	−1.8	Oroxylin A-7-*O*-*β*-D-glucuronide
28^*∗*^	19.93	C_22_H_20_O_11_	459.03219	459.09213	283.05975 (64.17), 268.03641 (100.00), 175.02324 (7.04), 163.00238 (5.15)	−0.1	Oroxindin
29^*∗*^	20.40	C_15_H_10_O_5_	269.04445	269.04443	251.03349 (2.73), 223.03867 (4.85)	−0.1	Baicalein
30^*∗*^	20.90	C_16_H_12_O_5_	283.06010	283.06003	268.03644 (100.00)	−0.2	Wogonin
31^*∗*^	20.96	C_16_H_12_O_5_	283.06010	283.06003	268.03644 (100.00), 239.03375 (4.28)	−0.2	Oroxylin A

^#^Adduct ion *m/z* [M + HCOOH–H]^–^. ^*∗*^Compared with the reference compounds.

**Table 2:** Collated results for linearity, LODs, LOQs, intra- and interday precisions, repeatability, stability, and recovery test of the 22 detected analytes in SHLI.

Analytes	Regression equations	*r*	Linear Range (*µ*g/mL)	LOQs (*µ*g/mL)	LODs (*µ*g/mL)	Precision		Repeatability (RSD, %, *n* = 6)	Stability (RSD, %, *n* = 6)	Recovery Test	
						Intraday (RSD, %; *n* = 6)	Interday (RSD, %; *n* = 3)			Recovery rate (%)	RSD (%)
Nea	*y* = 20099*x* − 428.07	0.999	0.5120–32.77	0.0569	0.0190	0.1	0.1	0.4	0.4	101.7	1.6
FoE	*y* = 1432.6*x* + 741.52	0.999	1.563–100.0	0.5208	0.1736	0.3	0.4	0.4	0.2	102.3	1.8
Cha	*y* = 21363*x* − 3658.0	0.999	0.6088–38.96	0.0676	0.0226	0.1	0.1	0.4	0.3	100.2	1.0
Cra	*y* = 9546.6*x* − 1111.6	0.999	0.5895–37.73	0.1965	0.0655	0.1	0.3	0.3	0.4	100.8	2.5
Caa	*y* = 15144*x* + 618.54	0.999	0.0945–6.048	0.0945	0.0315	0.7	3.4	1.5	2.7	105.4	1.9
Sea	*y* = 6601.9*x* + 461.97	0.999	0.5000–32.00	0.5000	0.1667	0.6	2.4	1.2	1.8	97.39	2.3
Seg	*y* = 11802*x* − 1412.3	0.999	0.2813–18.00	0.2813	0.0938	0.2	0.4	0.2	0.5	101.3	1.3
Iso	*y* = 10864*x* − 3542.2	0.999	1.167–74.66	0.1296	0.0432	0.1	0.1	0.4	0.3	102.2	1.7
Hyp	*y* = 10263 − 337.99	0.999	0.1037–6.636	0.1037	0.0346	0.3	0.4	0.4	0.7	102.7	1.0
Scu	*y* = 23346*x* − 2670.7	0.999	0.1813–11.60	0.0604	0.0202	0.2	0.2	1.0	0.5	109.3	1.3
FoA	*y* = 9905.5*x* − 3660.3	0.999	1.209–77.40	0.1344	0.0448	0.1	0.3	0.4	0.2	98.17	1.8
IaB	*y* = 20276*x* − 2334.8	0.999	0.2613–16.72	0.0871	0.0290	0.5	0.5	0.4	0.4	101.4	1.0
IaA	*y* = 24072*x* − 1409.8	0.999	0.09375–6.000	0.0938	0.0313	0.5	0.5	0.4	0.4	107.6	1.1
IaC	*y* = 23139*x* − 3039.9	0.999	0.2630–16.83	0.0877	0.0292	0.6	0.3	0.3	0.4	102.3	1.1
Pil	*y* = 4697.3*x* − 696.84	0.999	0.4345–27.81	0.4345	0.1448	1.0	0.3	0.8	0.7	95.06	2.3
Chg	*y* = 25004*x* − 2952.2	0.999	0.3444–22.04	0.1148	0.0383	0.1	0.2	0.4	0.2	98.76	2.7
Org	*y* = 23574*x* − 7113.5	0.999	0.8750–56.00	0.0972	0.0324	0.1	0.1	0.3	0.2	103.7	0.7
Oro	*y* = 29742*x* − 741.55	0.999	0.07225–4.624	0.0723	0.0241	0.5	0.5	1.2	0.7	103.1	2.0
Bai	*y* = 44049*x* − 6983.7	0.999	0.1581–10.12	0.1581	0.0527	0.3	3.5	1.5	1.0	107.5	2.2
Won	*y* = 46125*x* − 496.68	0.999	0.0147–0.9386	0.0147	0.0049	0.6	2.0	1.1	0.6	106.0	2.7
OrA	*y* = 48713*x* − 422.74	0.999	0.01291–0.8260	0.0129	0.0043	0.4	1.3	1.4	1.1	95.52	1.6
Bac	*y* = 26701*x* − 15617	0.999	1.875–120.0	0.0667	0.0222	0.1	0.2	0.5	0.3	100.7	3.0

**Table 3:** Comparation between the previously reported analytical methods and our established method.

Drugs	Analysis instrument	Analytes	Running time (min)
SHLI [26]	HPLC	FoA	35
SHLI [27]	HPLC	Lug	60
SHLI [28]	HPLC	Cha, Caa, Iac, Bac, Bai, and Won	50
SHLPI [29]	HPLC	Cha, Caa, FoA, Bac, and Pil	60
SHLPI [17]	HPLC	Nea, Cha, Cra, Caa, Iso, IaA, Lug, Rutin, Iac, Pil, Bac, Oro, Org, Bai, and Won	60
SHLPI [30]	UPLC	Cha, Bac, and Pil	20
SHLI^#^	UPLC	Nea, FoE, Cha, Cra, Caa, Sea, Seg, Iso, Hyp, Scu, FoA, IaB, IaA, IaC, Pil, Chg, Org, Oro, Bai, Won, OrA, and Bac	24

^#^The established method in our study.

## Data Availability

The data used to support the findings of this study are available from the corresponding author upon request.

## Supplementary Materials

Supplementary Materials
*Table S1*. All the targets corresponding to the 22 index compounds in SHLI. *Table S2*. The KEGG Pathway enrichment analysis results of all targets (*p* < 0.01). *Table S3*. The quantified result of 22 candidate compounds in SHLI of 20 batches (*n* = 2). *Table S4*. The percentage of tested components in total solid content in SHLI. *Figure S1*. The proposed fragmentation pathways of Seg in the negative mode. *Figure S2*. The proposed fragmentation pathways of Bac in the negative mode.
