# Supplementary material for: Integrating Study on Qualitative and Quantitative Characterization of the Major Constituents in Shuanghuanglian Injection with UHPLC/Q-Orbitrap-MS and UPLC-PDA
Source: J Anal Methods Chem. 2021 May 21;2021:9991363. doi: 10.1155/2021/9991363 (PMC8163532; doi:10.1155/2021/9991363)
Supplement: Supplementary Materials — Table S1. All the targets corresponding to the 22 index compounds in SHLI. Table S2. The KEGG Pathway enrichment analysis results of all targets (p < 0.01). Table S3. The quantified result of 22 candidate compounds in SHLI of 20 batches (n = 2). Table S4. The percentage of tested components in total solid content in SHLI. Figure S1. The proposed fragmentation pathways of Seg in the negative mode. Figure S2. The proposed fragmentation pathways of Bac in the negative mode. [file 9991363.f1.docx]

**Supplementary Materials to:**

**Integrating study on qualitative and quantitative characterization of the major constituents in Shuanghuanglian injection with UHPLC/Q-Orbitrap-MS and UPLC-PDA**

Table S1: All the targets corresponding to the 22 index compounds in SHLI.

| Targets | | | | | | | | | |
| --- | --- | --- | --- | --- | --- | --- | --- | --- | --- |
| AKR1B1 | ENGASE | AMY2A | MAPK1 | HSP90AA1 | PIM1 | ALDH2 | CDK5R1 | TOP1 | PRKDC |
| AKR1B10 | ECE1 | CA7 | AKR1C3 | ATIC | PTGDR2 | FDFT1 | CCNB3 | SRC | MAPK3 |
| MMP12 | OGA | CA4 | AKR1C4 | ALOX12 | POLB | CSNK1D | CDK6 | NAE1 | SIRT1 |
| APP | ELANE | CA13 | AKR1C2 | SLC5A2 | SELE | CYP19A1 | ABCG2 | AHR | PLA2G2A |
| MMP13 | KDR | CA5A | SYK | SELP | KDM3A | GABRA5 | CBR1 | ESRRA | PLA2G4A |
| MMP2 | CASP6 | HRAS | EGFR | LGALS3 | KDM4C | TDP1 | PTPRS | CYP1A1 | CXCR1 |
| SLC37A4 | CASP7 | PDE5A | FYN | NOD2 | TYMS | NTRK1 | AMY1A | KIT | OPRM1 |
| PYGL | CASP8 | EDNRA | LCK | IMPDH1 | NPC1L1 | MTOR | FLT3 | OPRD1 | MCL1 |
| PRKCD | CASP1 | MGLL | PTGS1 | IMPDH2 | SELL | SLC2A1 | IKBKB | AURKB | DAPK1 |
| PRKCA | CASP2 | PTPN22 | PIK3CB | NOX4 | GART | ICAM1 | NTRK2 | MAPT | MPG |
| CA2 | EGLN1 | ALOX5 | CYP1A2 | ADRA2C | MAP3K14 | GBA | AR | TOP2A | MPO |
| CA1 | ADAMTS5 | CA6 | CYP2C9 | ACHE | SLC5A1 | ADORA2B | MAOA | INSR | PIK3R1 |
| CA12 | FTO | MMP9 | CYP3A4 | RPS6KA3 | RAF1 | ALOX15 | GSK3B | MYLK | MMP3 |
| CA9 | DNMT3B | PTPN1 | CYP2C19 | NMUR2 | BRAF | BCL2A1 | ABCC1 | APEX1 | CAMK2B |
| NEU4 | TREH | CA3 | PIK3CA | ADRA2A | DHFR | GPR55 | CFTR | PIK3CG | PLA2G1B |
| BACE1 | TYR | ESR2 | F3 | PTGS2 | AMPD3 | IGFBP3 | TNKS2 | IGF1R | AKR1C1 |
| PDE4D | MMP1 | HCAR2 | HSD11B1 | CD38 | CSNK2A1 | SLC28A2 | TNKS | GPR35 | AKR1A1 |
| PDE9A | MMP7 | MIF | MAOB | TNF | THRA | MAPK14 | HSD17B2 | PLK1 | ODC1 |
| PDE1B | MMP8 | NQO2 | NFE2L2 | IL2 | THRB | HPRT1 | BCHE | MET | HSP90B1 |
| CASP3 | FUCA1 | TLR4 | STAT3 | XDH | LTB4R | KDM4E | PFKFB3 | ALK | AVPR2 |
| CA5B | CA14 | ERBB2 | ADORA1 | SLC29A1 | SLC46A1 | CDK1 | CALM1 | AXL | PLG |
| ABCB1 | ADORA2A | ESR1 | ADA | TERT | FOLR1 | GRK6 | NOS2 | ST6GAL1 | SIGMAR1 |
| NEU3 | ADORA3 | SLC6A2 | ADK | LGALS1 | SLC19A1 | HSD17B1 | ARG1 | SLC22A12 | TACR2 |
| NEU2 | SLC28A3 | TTR | FOLH1 | PARP1 | FOLR2 | CYP1B1 | GLO1 |  |  |

Table S2: The KEGG enrichment analysis results of all targets (*p* < 0.01).

| Term | | *p* value | Term | *p* value | Term | *p* value |
| --- | --- | --- | --- | --- | --- | --- |
| Nitrogen metabolism | 0.0000 | | Apoptosis | 0.0000 | Osteoclast differentiation | 0.0004 |
| HIF-1 signaling pathway | 0.0000 | | Sphingolipid signaling pathway | 0.0000 | cAMP signaling pathway | 0.0006 |
| Central carbon metabolism in cancer | 0.0000 | | Natural killer cell mediated cytotoxicity | 0.0000 | MicroRNAs in cancer | 0.0006 |
| Glioma | 0.0000 | | Insulin resistance | 0.0000 | Long-term potentiation | 0.0006 |
| Estrogen signaling pathway | 0.0000 | | Rap1 signaling pathway | 0.0000 | Fc gamma R-mediated phagocytosis | 0.0007 |
| Prostate cancer | 0.0000 | | Renal cell carcinoma | 0.0000 | NF-kappa B signaling pathway | 0.0009 |
| Acute myeloid leukemia | 0.0000 | | Insulin signaling pathway | 0.0000 | Toll-like receptor signaling pathway | 0.0009 |
| Proteoglycans in cancer | 0.0000 | | GnRH signaling pathway | 0.0000 | Toxoplasmosis | 0.0012 |
| TNF signaling pathway | 0.0000 | | Linoleic acid metabolism | 0.0000 | Oxytocin signaling pathway | 0.0013 |
| Pathways in cancer | 0.0000 | | Inflammatory mediator regulation of TRP channels | 0.0000 | Cholinergic synapse | 0.0013 |
| Non-small cell lung cancer | 0.0000 | | B cell receptor signaling pathway | 0.0000 | Long-term depression | 0.0016 |
| VEGF signaling pathway | 0.0000 | | Focal adhesion | 0.0000 | Tuberculosis | 0.0018 |
| Bladder cancer | 0.0000 | | Ras signaling pathway | 0.0000 | cGMP-PKG signaling pathway | 0.0020 |
| Serotonergic synapse | 0.0000 | | Hepatitis C | 0.0000 | Chemical carcinogenesis | 0.0021 |
| ErbB signaling pathway | 0.0000 | | Vascular smooth muscle contraction | 0.0000 | Melanogenesis | 0.0023 |
| Neurotrophin signaling pathway | 0.0000 | | Adherens junction | 0.0000 | Signaling pathways regulating pluripotency of stem cells | 0.0023 |
| Fc epsilon RI signaling pathway | 0.0000 | | Chronic myeloid leukemia | 0.0000 | Viral carcinogenesis | 0.0024 |
| Prolactin signaling pathway | 0.0000 | | Chagas disease (American trypanosomiasis) | 0.0000 | MAPK signaling pathway | 0.0026 |
| T cell receptor signaling pathway | 0.0000 | | PI3K-Akt signaling pathway | 0.0001 | Neuroactive ligand-receptor interaction | 0.0026 |
| Progesterone-mediated oocyte maturation | 0.0000 | | Colorectal cancer | 0.0001 | Metabolic pathways | 0.0029 |
| Endometrial cancer | 0.0000 | | Aldosterone-regulated sodium reabsorption | 0.0001 | Epithelial cell signaling in Helicobacter pylori infection | 0.0031 |
| FoxO signaling pathway | 0.0000 | | Other glycan degradation | 0.0001 | Alzheimer's disease | 0.0033 |
| Pancreatic cancer | 0.0000 | | Influenza A | 0.0001 | Amoebiasis | 0.0034 |
| Steroid hormone biosynthesis | 0.0000 | | Choline metabolism in cancer | 0.0001 | Gap junction | 0.0038 |
| mTOR signaling pathway | 0.0000 | | Carbohydrate digestion and absorption | 0.0002 | Purine metabolism | 0.0048 |
| Melanoman | 0.0000 | | NOD-like receptor signaling pathway | 0.0002 | Measles | 0.0050 |
| Hepatitis B | 0.0000 | | Regulation of lipolysis in adipocytes | 0.0002 | Calcium signaling pathway | 0.0055 |
| Type II diabetes mellitus | 0.0000 | | Metabolism of xenobiotics by cytochrome | 0.0003 | Tryptophan metabolism | 0.0056 |
| Thyroid hormone signaling pathway | 0.0000 | | Pertussis | 0.0003 | Epstein-Barr virus infection | 0.0086 |
| Ovarian steroidogenesis | 0.0000 | | Chemokine signaling pathway | 0.0003 | AMPK signaling pathway | 0.0091 |
| Arachidonic acid metabolism | 0.0000 | | Platelet activation | 0.0004 | Thyroid cancer | 0.0092 |

Table S3: The quantified result of 22 candidate compounds in SHLI of 20 batches (*n*=2).

| Batches  Compounds | Content (μg/mL) | | | | | | | | | |
| --- | --- | --- | --- | --- | --- | --- | --- | --- | --- | --- |
|  | S1 | S2 | S3 | S4 | S5 | S6 | S7 | S8 | S9 | S10 |
| Nea | 228.0 | 236.2 | 254.8 | 239.1 | 270.5 | 244.7 | 224.2 | 229.0 | 237.5 | 207.6 |
| FoE | 560.4 | 560.2 | 762.4 | 609.3 | 793.2 | 626.0 | 717.9 | 640.1 | 668.0 | 617.1 |
| Cha | 178.4 | 187.0 | 198.5 | 184.8 | 210.6 | 195.7 | 179 | 186.1 | 193.5 | 169.2 |
| Cra | 217.0 | 224.8 | 246.5 | 226.8 | 259.9 | 231.5 | 213.1 | 217.0 | 224.3 | 202.0 |
| Caa | 46.70 | 46.56 | 53.26 | 50.23 | 61.10 | 48.25 | 66.88 | 56.41 | 57.54 | 63.48 |
| Sea | 348.1 | 348.3 | 582.3 | 476.7 | 542.1 | 414.8 | 365.6 | 316.2 | 352.5 | 362.4 |
| Seg | 88.82 | 96.08 | 111.7 | 87.76 | 88.22 | 77.84 | 75.29 | 74.88 | 85.19 | 85.38 |
| Iso | 385.9 | 393.8 | 454.4 | 383.6 | 508.7 | 427.8 | 484.0 | 456.8 | 486.2 | 467.1 |
| Hyp | 66.77 | 64.80 | 67.21 | 67.25 | 89.45 | 64.99 | 72.55 | 67.73 | 70.16 | 80.49 |
| Scu | 56.29 | 58.28 | 52.87 | 50.17 | 71.55 | 84.30 | 83.79 | 81.69 | 76.54 | 70.72 |
| FoA | 425.3 | 438.3 | 503.4 | 419.0 | 560.6 | 480.6 | 546.9 | 520.1 | 553.0 | 531.2 |
| IaB | 78.87 | 104.3 | 101.2 | 91.54 | 95.39 | 97.66 | 98.19 | 92.50 | 101.7 | 79.69 |
| IaA | 23.93 | 31.06 | 29.99 | 27.53 | 27.93 | 29.68 | 30.56 | 28.96 | 31.91 | 24.68 |
| IaC | 101.6 | 132.5 | 128.4 | 119.3 | 120.5 | 121.6 | 118.3 | 110.1 | 120.4 | 93.02 |
| Pil | 161.8 | 157.2 | 172.1 | 146.0 | 183.9 | 168.0 | 163.5 | 159.0 | 172.2 | 158.5 |
| Chg | 138.5 | 142.4 | 135.6 | 138.6 | 130.8 | 137.8 | 128.4 | 131.8 | 128.0 | 127.1 |
| Org | 354.9 | 359.0 | 369.3 | 360.8 | 313.5 | 343.9 | 316.3 | 312.9 | 291.2 | 328.9 |
| Oro | 22.58 | 17.39 | 23.27 | 17.39 | 20.27 | 14.77 | 14.71 | 19.87 | 14.63 | 20.81 |
| Bai | 69.97 | 63.31 | 86.26 | 97.58 | 99.79 | 108.9 | 90.51 | 70.72 | 84.67 | 94.22 |
| Won | 2.750 | 2.765 | 3.104 | 3.493 | 4.670 | 4.595 | 2.400 | 2.191 | 2.403 | 3.710 |
| OrA | 3.417 | 3.813 | 3.962 | 4.180 | 3.666 | 4.933 | 3.677 | 3.046 | 2.990 | 3.773 |
| Bac | 6692 | 6755 | 6578 | 6649 | 6823 | 6839 | 6554 | 6464 | 6553 | 6568 |
| Total* | 10.26 | 10.42 | 10.92 | 10.46 | 11.28 | 10.77 | 10.55 | 10.24 | 10.51 | 10.36 |
| *, The content unit is mg/mL | | | | | | | | | | |

Table S3: (Continued) The quantified result of 22 candidate compounds in SHLI of 20 batches (*n*=2).

| Batches  Compounds | Content (μg/mL) | | | | | | | | | |
| --- | --- | --- | --- | --- | --- | --- | --- | --- | --- | --- |
|  | S11 | S12 | S13 | S14 | S15 | S16 | S17 | S18 | S19 | S20 |
| Nea | 233.7 | 228.0 | 227.6 | 208.3 | 247.4 | 212.3 | 217.4 | 200.8 | 190.4 | 212.8 |
| FoE | 581.3 | 477.9 | 535.4 | 598.7 | 726.5 | 687.1 | 699.4 | 633.3 | 545.4 | 655.1 |
| Cha | 194.2 | 194.1 | 195.8 | 179.1 | 210.8 | 181.9 | 185.2 | 165.6 | 187.6 | 207.0 |
| Cra | 230.2 | 231.9 | 222.2 | 207.9 | 243.9 | 212.0 | 211.8 | 194.4 | 206.1 | 234.5 |
| Caa | 51.46 | 39.52 | 50.12 | 50.37 | 52.31 | 65.69 | 77.59 | 91.52 | 61.31 | 72.05 |
| Sea | 508.5 | 398.3 | 456.5 | 439.8 | 607.8 | 525.0 | 358.0 | 364.1 | 306.4 | 492.9 |
| Seg | 140.4 | 130.3 | 96.07 | 140.7 | 116.0 | 89.17 | 76.54 | 66.75 | 71.16 | 100.6 |
| Iso | 424.9 | 364.0 | 451.6 | 484.8 | 528.4 | 533.6 | 506.0 | 410.6 | 554.7 | 642.4 |
| Hyp | 64.49 | 55.83 | 76.32 | 78.23 | 71.30 | 77.53 | 78.06 | 72.36 | 80.58 | 84.23 |
| Scu | 92.29 | 69.15 | 67.22 | 96.38 | 84.09 | 85.07 | 68.9 | 86.32 | 52.04 | 55.79 |
| FoA | 490.3 | 421.7 | 521.0 | 557.7 | 611.6 | 617.9 | 585.8 | 475.0 | 649.1 | 749.9 |
| IaB | 109.4 | 109.2 | 102.7 | 92.53 | 99.49 | 102.5 | 90.47 | 92.57 | 89.69 | 110.6 |
| IaA | 33.24 | 35.40 | 33.28 | 30.26 | 33.24 | 35.09 | 33.32 | 29.58 | 41.93 | 52.37 |
| IaC | 123.4 | 123.9 | 114.5 | 103.5 | 108.2 | 110.8 | 94.56 | 99.32 | 87.60 | 105.1 |
| Pil | 161.2 | 155.9 | 157.2 | 162.7 | 158.5 | 169.2 | 153.9 | 140.9 | 146.1 | 160.9 |
| Chg | 137.6 | 134.2 | 130.7 | 131.7 | 129.1 | 123.7 | 129.4 | 124.7 | 131.1 | 133.6 |
| Org | 333.0 | 357.4 | 341.2 | 311.1 | 330.6 | 305.3 | 331.4 | 302.0 | 357.7 | 361.9 |
| Oro | 19.63 | 17.39 | 15.26 | 16.66 | 20.26 | 16.18 | 18.97 | 13.06 | 15.36 | 18.99 |
| Bai | 72.47 | 55.03 | 69.04 | 64.90 | 69.20 | 86.69 | 72.68 | 84.92 | 57.68 | 66.66 |
| Won | 3.740 | 3.301 | 3.530 | 3.194 | 2.098 | 2.822 | 2.663 | 3.611 | 2.199 | 2.533 |
| OrA | 3.935 | 4.397 | 4.131 | 3.783 | 3.450 | 3.215 | 3.483 | 3.653 | 3.684 | 3.786 |
| Bac | 6559 | 6646 | 6597 | 6681 | 6506 | 6765 | 6611 | 6881 | 6679 | 6696 |
| Total* | 10.57 | 10.26 | 10.47 | 10.64 | 10.96 | 11.01 | 10.61 | 10.54 | 10.52 | 11.22 |
| *, The content unit is mg/mL | | | | | | | | | | |

Table S4: The percentage of tested components in total solid content in SHLI.

| Batches | Batch No. | Total solid  (mg/per ampoule) | Total content of the tested components (mg/per ampoule) | Percentage  (%) |
| --- | --- | --- | --- | --- |
| S1 | 1801253 | 549.3 | 205.2 | 37.4 |
| S2 | 1802051 | 534.4 | 208.4 | 39.0 |
| S3 | 1803051 | 566.1 | 218.4 | 38.6 |
| S4 | 1804281 | 561.9 | 209.2 | 37.2 |
| S5 | 1805111 | 588.8 | 225.6 | 38.3 |
| S6 | 1806293 | 520.9 | 215.4 | 41.4 |
| S7 | 1807161 | 504.9 | 211.0 | 41.8 |
| S8 | 1808031 | 549.9 | 204.8 | 37.2 |
| S9 | 1809031 | 532.3 | 210.2 | 39.5 |
| S10 | 1810023 | 536.0 | 207.2 | 38.7 |
| S11 | 1901021 | 540.5 | 211.4 | 39.1 |
| S12 | 1902051 | 510.6 | 205.2 | 40.2 |
| S13 | 1903091 | 556.0 | 209.4 | 37.7 |
| S14 | 1904051 | 561.0 | 212.8 | 37.9 |
| S15 | 1905081 | 613.2 | 219.2 | 35.7 |
| S16 | 1906061 | 590.4 | 220.2 | 37.3 |
| S17 | 1907022 | 552.6 | 212.2 | 38.4 |
| S18 | 1908011 | 521.3 | 210.8 | 40.4 |
| S19 | 1910052 | 532.0 | 210.6 | 39.6 |
| S20 | 1911023 | 579.5 | 224.4 | 38.7 |
| Average | – | – | – | 38.7 |


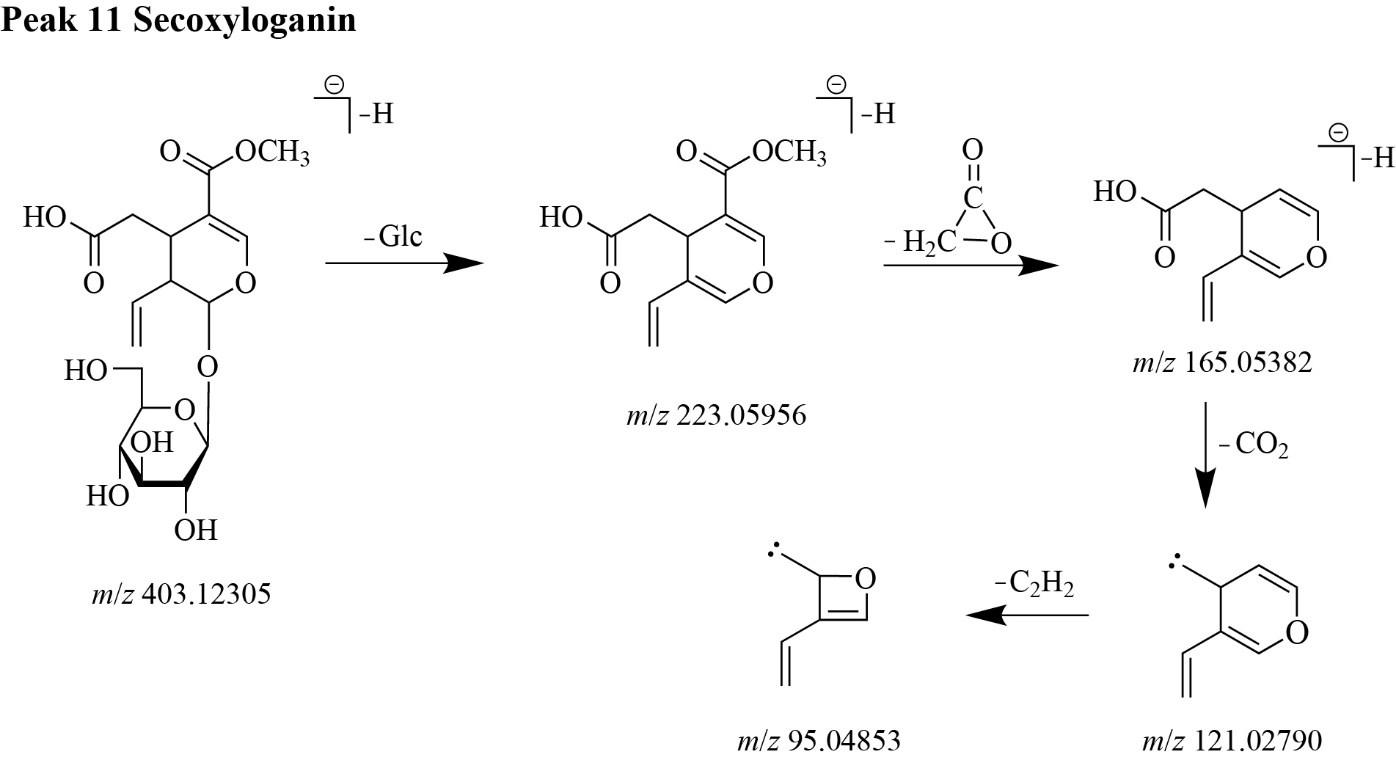


Figure S1: The proposed fragmentation pathways of Seg in the negative mode.


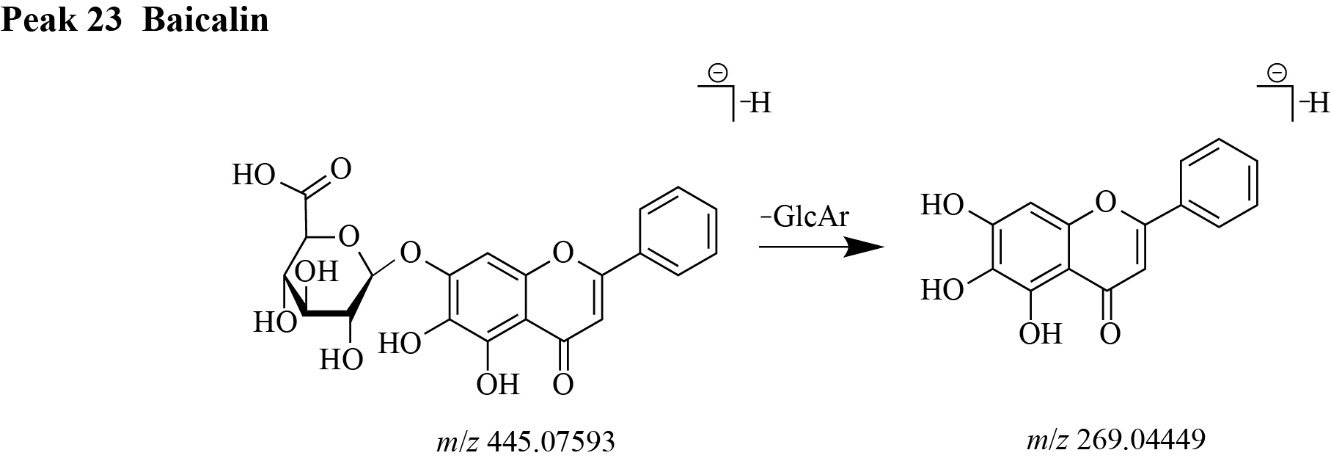


Figure S2: The proposed fragmentation pathways of Bac in the negative mode.
